# JADE: jawbone lesion diagnosis and decision supporting system

**DOI:** 10.1093/dmfr/twag017

**Published:** 2026-03-23

**Authors:** Soroush Baseri Saadi, Jonas Ver Berne, Rocharles Cavalcante Fontenele, Peter Claes, Reinhilde Jacobs

**Affiliations:** OMFS-IMPATH Research Group, Department of Imaging and Pathology, Catholic University Leuven, Leuven 3000, Belgium; Department of Oral and Maxillofacial Surgery, University Hospitals Leuven, Leuven 3000, Belgium; OMFS-IMPATH Research Group, Department of Imaging and Pathology, Catholic University Leuven, Leuven 3000, Belgium; Department of Pathology, University Hospitals Leuven, Leuven 3000, Belgium; OMFS-IMPATH Research Group, Department of Imaging and Pathology, Catholic University Leuven, Leuven 3000, Belgium; Division of Oral Radiology, Department of Stomatology, Public Health, and Forensic Dentistry, School of Dentistry of Ribeirao Preto, University of Sao Paulo (USP), Ribeirao Preto, Sao Paulo 14040-904, Brazil; Department of Human Genetics, KU Leuven, Leuven 3000, Belgium; Medical Imaging Research Center, UZ Leuven, Leuven 3000,Belgium; Department of Electrical Engineering, Processing of Speech and Images (ESAT-PSI), KU Leuven, Leuven 3000, Belgium; OMFS-IMPATH Research Group, Department of Imaging and Pathology, Catholic University Leuven, Leuven 3000, Belgium; Department of Oral and Maxillofacial Surgery, University Hospitals Leuven, Leuven 3000, Belgium; Department of Dental Medicine, Karolinska Institutet, Stockholm SE-141 04, Sweden

**Keywords:** jawbone lesions, differential diagnosis, large language models, retrieval-augmented generation, cloud-based applications, hybrid retrieval

## Abstract

**Objectives:**

To develop and evaluate JADE, a proof-of-concept retrieval-augmented generation (RAG) diagnostic assistive system, designed to enhance large language model (LLM) reasoning for jawbone lesion assessment. This study examined whether RAG improves diagnostic accuracy and stability compared with standalone LLMs and ORAD, a supervised learning-based system.

**Methods:**

JADE was developed as a cloud-based RAG system integrating an expert-curated oral radiology database embedded using text-embedding-3-large and indexed in Qdrant Cloud. Structured clinical inputs were encoded as prioritized vector queries. Hybrid semantic and keyword-based retrieval appended relevant evidence to prompts for differential diagnosis generation across LLM backbones. Performance was evaluated in 25 validation cases and compared with GPT-5, Claude Sonnet 4.5, DeepSeek-R1, Gemini 2.5 Flash, their RAG configurations, and ORAD. Diagnostic accuracy was analyzed using Cochran’s *Q* test, with post-hoc McNemar’s tests and Bonferroni correction. Intra-model stability and response time were assessed.

**Results:**

RAG-GPT-5 achieved the highest diagnostic accuracy (20/25), followed by RAG-Claude Sonnet 4.5 (18/25), RAG-DeepSeek R1 (17/25), and RAG-Gemini 2.5 Flash (15/25). Standalone models achieved 9–13/25 correct diagnoses, ORAD achieved 17/25. No significant differences were observed among standalone models or RAG-based models and ORAD. A significant improvement was observed for GPT-5 when integrated with RAG (*P *= .002). RAG configurations showed higher intra-model stability, with RAG-GPT-5 achieving mean stability of 0.90 ± 0.11. Mean response times ranged from 3 to 10 s.

**Conclusions:**

JADE improved diagnostic accuracy and stability compared with standalone LLMs, underscoring value of RAG reasoning in jawbone lesion assessment and marking the first RAG application in dentomaxillofacial radiology.

## Introduction

Diagnosis of jawbone lesions is a highly challenging task in clinical practice, as these lesions have diverse origins, unique anatomical structures, and often overlapping radiographic characteristics.^1^ Even though some lesions may be treated similarly, ranging from medication to surgical intervention, accurate differentiation and diagnosis are necessary to avoid unnecessary invasive procedures or inappropriate medical treatments.^2^

Panoramic radiography is commonly used in daily dental practice as the main imaging modality for diagnosis, as it provides a broad 2D view of the dentomaxillofacial structures.^3^ However, interpreting panoramic radiographs is quite a complex task, especially for a general dentist who often lacks the knowledge of an oral radiologist. Besides, considering the global shortage of oral radiologists, the diagnostic process can be time-consuming and subject to error.

Artificial intelligence (AI) has demonstrated great potential in medical diagnosis in oral healthcare. Deep learning (DL), a subfield of AI, in combination with image data, has shown promising results in the detection and classification of jawbone lesions on panoramic radiographs.^4^^,^^5^ However, precise diagnosis of jawbone lesions is beyond image analysis and requires the incorporation of multiple clinical and radiographic parameters.

Large language models (LLMs), as text-based generative AI systems, have demonstrated strong contextual reasoning abilities in dentomaxillofacial radiology,^6–8^ allowing them to interpret various clinical parameters and generate clinically relevant diagnostic reasoning. Advanced LLMs, such as OpenAI’s GPT series,^9^^,^^10^ DeepSeek,^9^^,^^11^ and Gemini,^12^^,^^13^ have further shown potential for decision support in oral and maxillofacial radiology. However, existing studies in this field have primarily focused on benchmarking standalone LLMs’ performance, relying only on the models’ pretraining knowledge. Consequently, responses may appear coherent and fluent but contain inaccuracies or fabricated information, commonly referred to as hallucinations.^14–17^ This limitation reduces their reliability as clinical decision-support tools and highlights a critical gap in their application to the differential diagnosis of jawbone lesions.

To date, no study has investigated whether the diagnostic reasoning power of LLMs in oral and maxillofacial radiology can be improved by extending their knowledge with domain-specific information beyond their general training. In particular, the potential of the retrieval-augmented generation (RAG) framework,^18–21^ which can enhance LLMs’ reasoning with external, up-to-date information, has not been explored for assessing jawbone lesions. While recent systematic reviews have reported the growing application of RAG systems in dentistry and broader healthcare settings,^22–24^ their application in oral and maxillofacial radiology has not yet been investigated.

Therefore, this proof-of-concept study aimed to develop and evaluate JADE (Jawbone Artificial intelligence Diagnostic Engine), a novel RAG-based decision-support system designed as a cloud-based, mobile-adapted application to support the differential diagnosis of jawbone lesions. The primary objective was to investigate whether the addition of a structured RAG framework improves diagnostic performance and intra-model stability compared with standalone LLMs across different model families. Additionally, the framework was compared with ORAD,^25^ a well-established computer-assisted differential diagnosis system in oral and maxillofacial radiology that is based on supervised learning.

## Methods

### Ethical concerns

The retrospective study was carried out at the Centre of Dentomaxillofacial Radiology and the Department of Oral and Maxillofacial Surgery, University Hospitals Leuven, Leuven, Belgium. The Institutional Ethics Committee approved the study protocol (approval number: S65708), and all procedures adhered to the World Medical Association Declaration of Helsinki and the Institutional Review Board. Patient data were anonymized before analysis to ensure data protection and confidentiality. Moreover, no personal identifiers or metadata were used in this study beyond the radiographic and diagnostic information.

### JADE system architecture

**Figure 1:**
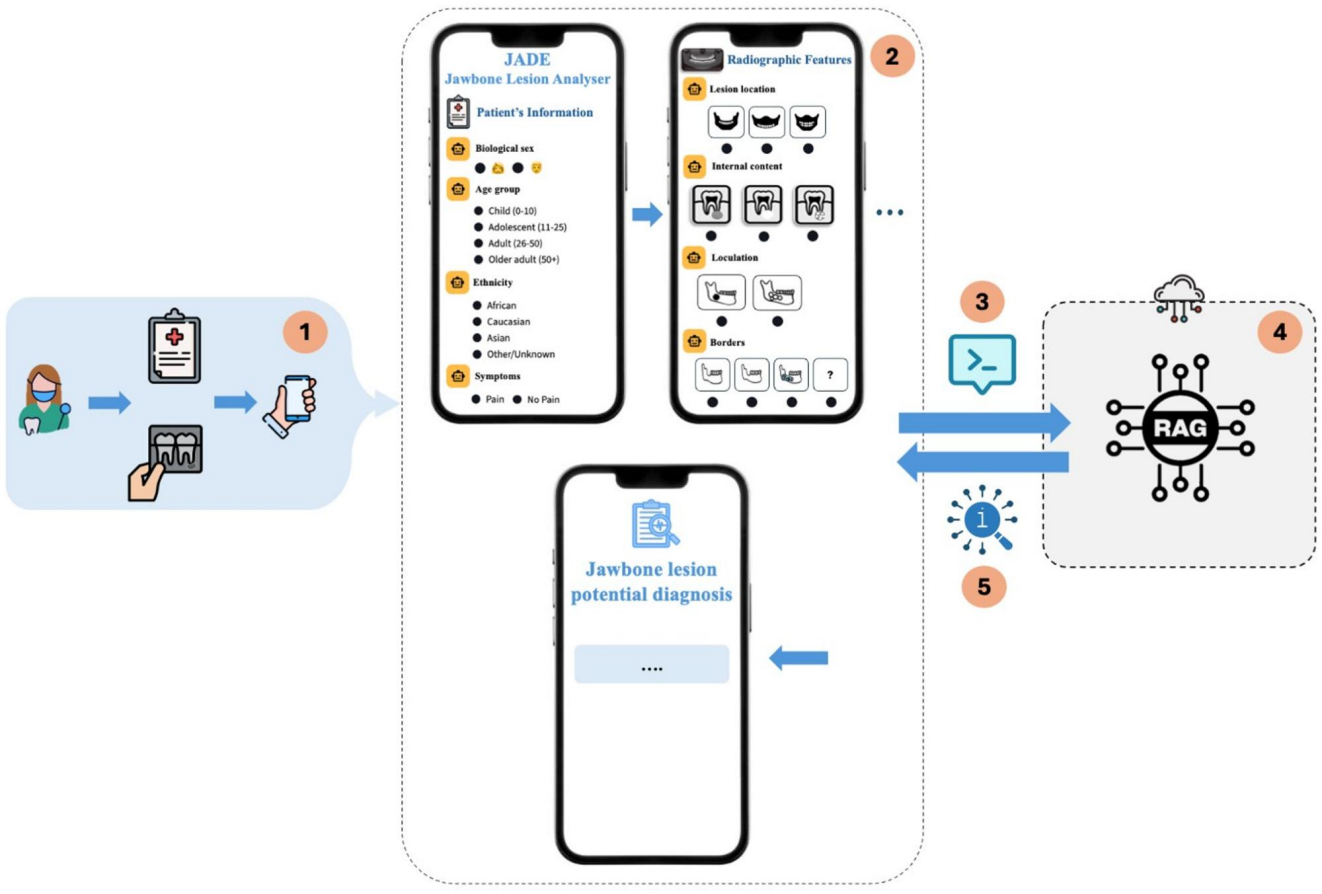
Overview of the JADE diagnostic workflow. (1) The clinician reviews the panoramic radiograph and the patient’s information, then enters the radiographic characteristics of the jawbone lesion together with relevant patient data into the application. (2) The application interface organizes these inputs into a structured and prioritized diagnostic query. (3) A formatted prompt is automatically generated, including reasoning instructions that define the diagnostic priority of each parameter. (4) The prompt is transmitted to the RAG module, where relevant domain-specific knowledge is retrieved to support the model’s diagnostic reasoning. (5) The final diagnostic response is generated and returned to the application interface as a structured differential diagnosis.

The JADE system was designed as a RAG-based diagnostic framework that integrated structured clinical input, domain-specific knowledge retrieval, and LLM-based reasoning within a unified system architecture (Figure 1).

Within this framework, the LLM functioned as the generative component responsible for producing the final differential diagnosis based on the structured clinical input, together with the retrieved domain-specific evidence. The model did not perform independent database searches; instead, it generated diagnostic output based on the contextual information supplied by the retrieval pipeline.

The RAG module relied on 2 complementary sources of information. The primary source consisted of a curated oral radiology and pathology database developed for this study by experienced oral and maxillofacial radiologists and pathologists. This database included detailed text-based descriptions of jawbone lesions derived from authoritative standard reference works in oral and maxillofacial radiology and pathology.^3^^,^^26–30^ The material was reformulated and standardized for research purposes and was formatted as a structured PDF document without the inclusion of images.

The document systematically described the most common lesions affecting the jawbones and organized them into 5 major categories: non-odontogenic bone lesions, odontogenic tumors, cysts of the jawbones, infectious and inflammatory bone pathology, and systemic diseases affecting the jaws. For each lesion, a structured description was provided, including an overview and definition, prevalence and epidemiology, clinical presentation, radiographic features on panoramic imaging, and radiological differential diagnosis. To ensure compliance with copyright regulations of the referenced books, only the list of included lesions and a representative example of the standardized lesion description format were provided in the Supplementary Material.

For vector database construction, the domain-specific database was embedded using text-embedding-3-large (chunk size = 1200, overlap = 200) and stored in a Qdrant Cloud vector database.^31^ In addition to this domain-specific knowledge source, the system also relied on the internal reasoning capability of the evaluated LLMs, which provided general medical knowledge based on their training data. The static knowledge cutoff varied depending on the specific model used during validation: GPT-5 (April 2025), Claude Sonnet 4.5 (August 2025), DeepSeek-R1 (May 2025), and Gemini 2.5 Flash (January 2025). This internal knowledge primarily supported diagnostic reasoning when the RAG failed to retrieve information from the domain-specific knowledge source.

The diagnostic process started with the clinician entering the answers to a set of ordered, predefined questions that recorded the patient information and the lesion’s radiographic characteristics through the application interface. These inputs were automatically arranged into a structured and prioritized query that formed the core of the model’s prompt (Table 1). The reasoning step was guided using the following prompt format:

**Table 1:** Prioritized patient’s information and radiographic questions were used to structure the diagnostic prompt.^a^

Question	Potential answers	Priority
Internal content	Radiolucent; radiopaque; mixed	High
Borders	Corticated; well-defined (non-corticated); diffuse; unknown	High
Loculation	Unilocular; multilocular	High
Relation with surrounding teeth	At the apex of a vital tooth; at the apex of a non-vital tooth; in contact with/involving root; in contact with crown; located in a missing-tooth area; not related to any tooth	High
Lesion location	Mandible; maxilla; both	High
Cortical expansion	Yes; no	High
Root resorption	Yes; no	High
Number of lesions	1; 2; ≥3; generalized	High
Biological sex	Male; female	Medium
Age group	Child (0-10); adolescent (11-25); adult (26-50); older adult (50+)	Medium
Ethnicity	African; Caucasian; Asian; other/unknown	Medium
Regional location	Incisor region; canine/premolar region; molar region; Ramus; TMJ; maxillary sinus; unknown	Medium
Symptoms	Pain; no pain	Medium
Lesion size	<2 cm; 2-3 cm; >3 cm; unknown	Low
Lesion includes 1 tooth	Yes; no	Low
Tooth displacement or impaction	Yes; no	Low

a The table outlines the set of standardized questions, their corresponding answer options, and their assigned priority levels, which collectively guide the system’s reasoning process in generating differential diagnoses.

“You are an oral radiologist. Provide the differential diagnosis for a jawbone lesion based on the following patient’s information and radiographic characteristics”

The prompt also included an instruction section that defined the diagnostic importance of each feature category. As indicated in Table 1, high-priority features consisted of core radiographic characteristics that directly influenced lesion classification, medium-priority features included demographic and contextual factors that provided supportive diagnostic information, and low-priority parameters represented additional features that refined but did not determine the final diagnosis. This structured format ensured that the model considered the most clinically meaningful features first, as well as supporting information properly, and kept a consistent diagnostic reasoning pathway across all cases.

After prompt construction, the structured query was encoded into a vector representation using the same text-embedding-3-large model used during vector database construction. The resulting query embedding was used to perform similarity matching against the indexed domain-specific database. JADE employed a hybrid retrieval approach that combined semantic (dense) and keyword-based (sparse) search to ensure both conceptual and keyword relevance.

Semantic (dense) retrieval used embedding similarity to identify text chunks that were conceptually similar to the query, even when different terminology or phrasing was used. For example, a description of a “multilocular radiolucent mandibular lesion in a young adult” retrieved conceptually related cases such as ameloblastoma or odontogenic keratocyst. Keyword (sparse) retrieval was based on BM25,^32^ a classical information-retrieval algorithm that ranked documents according to how often a term appeared and how informative or distinctive that term was within the dataset, while also accounting for document length. BM25 ensured that chunks containing explicitly mentioned terms (eg, “mandible,” “multilocular,” “radiolucent”) were reliably captured. Qdrant automatically merged dense and sparse results with its built-in hybrid score fusion, where both similarity signals were normalized and combined into a single relevance score. This default fusion logic ensured that neither semantic similarity nor exact keyword matching dominated, allowing conceptually aligned and lexically precise passages to be ranked appropriately.

**Figure 2:**
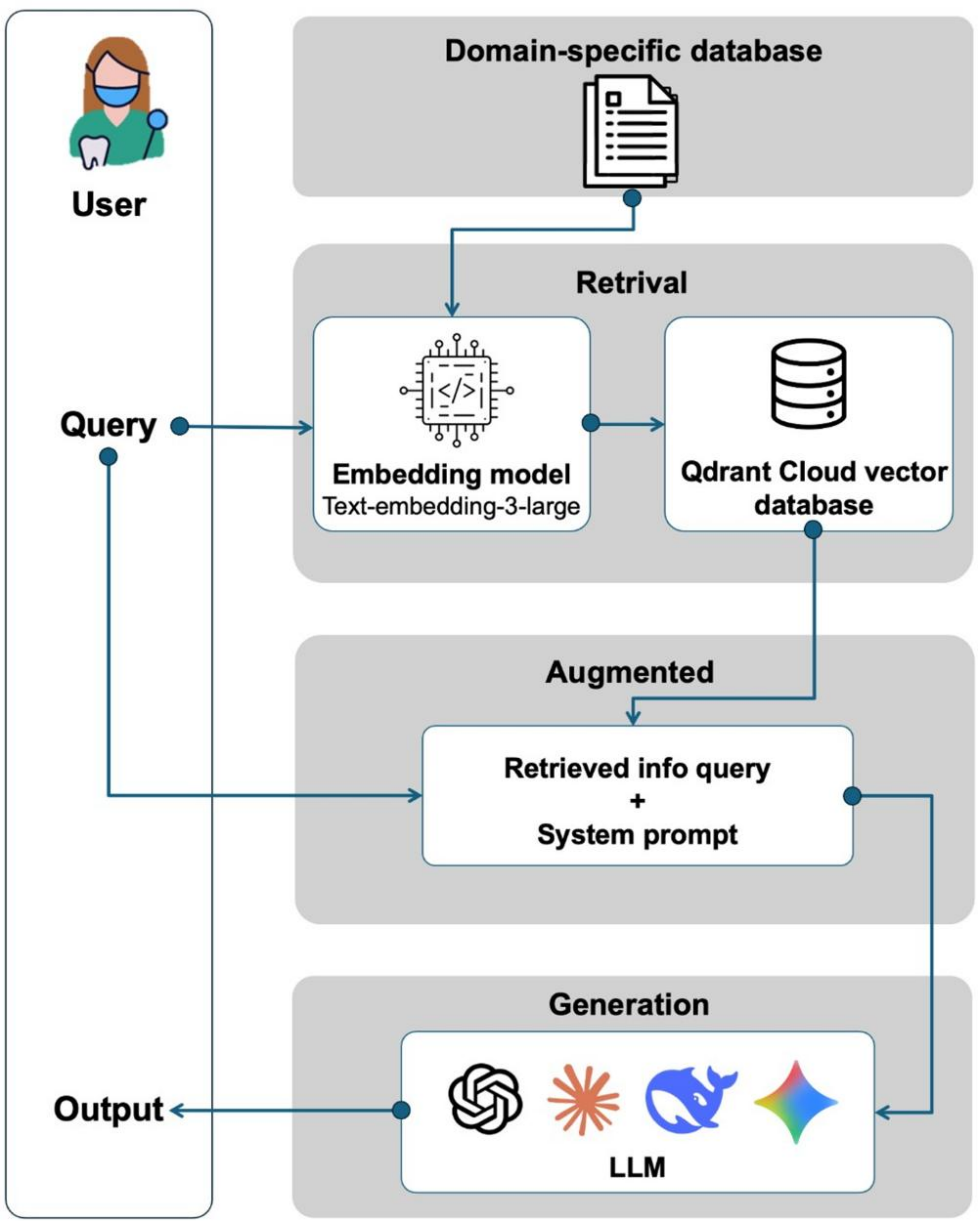
Internal architecture of the RAG module of JADE. The structured clinical query is encoded into a vector representation and used to retrieve relevant information from the indexed domain-specific oral radiology and pathology database stored in Qdrant Cloud. Retrieval is performed using a hybrid approach that combines semantic (dense) and keyword-based (BM25) search. The top-ranked retrieved text chunks are appended to the structured prompt as contextual evidence and provided to the LLM, which generates the differential diagnosis based on the retrieved domain-specific information, supplemented by its internal general medical knowledge when necessary.

For generations, the top-ranked retrieved chunks were appended to the structured prompt as contextual evidence and provided as input to the LLM. The LLM then generated the differential diagnosis by grounding its output in the retrieved domain-specific information, while using its internal general medical knowledge to support reasoning when retrieved evidence was limited or incomplete. The final output, representing the most probable differential diagnosis, was then sent to the system interface for display. Figure 2 demonstrates the internal workflow of the RAG module from query encoding to grounded response generation.

### Experimental setup and deployment

The application was developed in Python 3.12.10 and built using Streamlit,^33^ an open-source library for interactive web interfaces. To ensure reproducibility and environment portability, the application was containerized with Docker^34^ and deployed via Google Cloud Run,^35^ allowing automatic scaling in response to usage demand.

For model development and testing, computations were performed locally on a workstation equipped with a 12th Gen Intel Core i9-12900K processor operating at 3.20 GHz, 128 GB of RAM, and an NVIDIA RTX A5000 GPU with 24 GB of GDDR6 memory. The software environment ran on Microsoft Windows 11.

### Validation dataset and image acquisition

The retrospective validation dataset consisted of 25 representative cases: radicular cyst (*n* = 3), fibrous dysplasia (*n* = 2), Stafne bone defect (*n* = 2), dentigerous cyst (*n* = 4), ameloblastoma (*n* = 2), cemento-osseous dysplasia (*n* = 3), central giant cell granuloma (*n* = 2), Odontogenic Keratocyst (*n* = 4), and simple bone cyst (*n* = 3). The cohort included 12 male and 13 female patients with a mean age of 39 years (range 9-66 years). The majority of patients were European (*n* = 22), with 1 Asian patient and 2 African patients.

Two oral radiologists and pathologists selected the cases based on their frequency and pathological relevance. Cases were selected if their panoramic radiographs presented at least 1 jawbone lesion without any artifacts or distortions affecting diagnostic evaluation.

All panoramic radiographs were obtained digitally using either the VistaPano S (Dürr Dental, Bietigheim-Hissingen, Germany; 2013—current; 73 kV, 12 mA, 7 s) or the Promax 2D (Planmeca, Helsinki, Finland; 2013—current; 68 kV, 10 mA, 16 s). All imaging was performed by licensed dental radiology technicians with at least 3 years of clinical experience.

All included cases were confirmed by either expert-based or histopathological diagnosis. In cases of disagreement, a mutual agreement was reached through discussion to ensure the accuracy and reliability of the dataset.

### Models’ evaluation and statistical analysis

The diagnostic performance was evaluated against the ground truth reference diagnoses in the retrospective validation dataset. Initially, the standalone versions of the LLMs, including GPT-5, DeepSeek-R1, Claude Sonnet 4.5, and Gemini 2.5 Flash, were evaluated independently without retrieval augmentation. Subsequently, the RAG-based system was implemented locally, using the same LLMs as generative backbones, and the retrieval-augmented versions were evaluated under similar conditions. The results were further compared with ORAD, a supervised learning-based computer-assisted differential diagnosis system.

All models (standalone LLMs, RAG-augmented LLMs, and ORAD) were assessed based on their ability to generate a single most probable diagnosis, which was then compared with the corresponding reference diagnosis in the validation dataset. Diagnostic performance and model stability were quantified using the following metrics.

Proportion of correct diagnosis: This metric represents the proportion of validation cases for which the model’s predicted diagnosis correctly matched the reference diagnosis


$$\mathrm{Proportion}\;\mathrm{of}\;\mathrm{correct}\;\mathrm{diagnosis}=\frac{\mathrm{Number}\;\mathrm{of}\;\mathrm{correctly}\;\mathrm{diagnosed}\;\mathrm{cases}}{\mathrm{Total}\;\mathrm{number}\;\mathrm{of}\;\mathrm{cases}}$$

Majority agreement ratio (MAR): MAR assesses intra-model stability by evaluating how consistent a model is in producing the same diagnosis across multiple independent runs for each case *i*. A maximum of 10 runs per case was performed


$${\mathrm{MAR}}_{i}=\frac{\mathrm{Frequency}\;\mathrm{of}\;\mathrm{the}\;\mathrm{most}\;\mathrm{common}\;\mathrm{prediction}}{\mathrm{Number}\;\mathrm{of}\;\mathrm{runs}}$$

A higher MAR value means greater prediction consistency, regardless of whether the prediction is correct. Therefore, MAR reflects model stability, not diagnostic accuracy.

Mean stability: Overall stability of the model over the entire validation dataset


$$\mathrm{Mean}\;\mathrm{stabiltiy}=\frac{1}{N}\sum_{i}{\mathrm{MAR}}_{i}$$

where *N* = 25 validation cases.

Diagnostic performance of the standalone LLMs was statistically evaluated using Cochran’s *Q* test (Statsmodels, Python^36^) to compare differences in binary outcomes (1 = correct, 0 = incorrect) across related cases. Degrees of freedom were calculated as *k *− 1, where *k* represented the number of standalone models included in the comparison. The null hypothesis was that no differences in diagnostic accuracy existed among the models. If the overall test was statistically significant, pairwise McNemar exact tests were calculated to identify specific differences between models. The null hypothesis for each pairwise comparison was that no difference in diagnostic accuracy existed between the 2 models. A Bonferroni correction was applied to adjust for multiple comparisons. For the standalone model’s comparison (4 models), 6 pairwise comparisons were performed (4 × 3/2), resulting in an adjusted per-test significance threshold of *P *< .008 (.05/6), while maintaining a family-wise significance level of *P *< .05. *P*-values falling between these 2 thresholds were considered statistically suggestive but not significant after correction.

The same procedure was independently applied to the RAG-augmented LLMs and ORAD. As 5 model configurations were compared (4 RAG models plus ORAD), 10 pairwise comparisons were performed (5 × 4/2). Therefore, the Bonferroni-adjusted per-comparison significance threshold was set at *P *< .005 (0.05/10).

In addition, direct paired comparisons between each standalone LLM and its corresponding RAG configuration were performed using McNemar’s exact test, as these comparisons involved paired binary outcomes on the same validation cases. The null hypothesis was that no difference in diagnostic accuracy existed between each standalone model and its corresponding RAG implementation.

Furthermore, the mean ± SD response time of the RAG models implemented with different LLM backbones was calculated by recording latency over 10 independent inference runs for each of the 25 validation cases to evaluate computational efficiency. All measurements were carried out under stable network and hardware conditions to ensure consistent timing outcomes.

## Results

**Figure 3:**
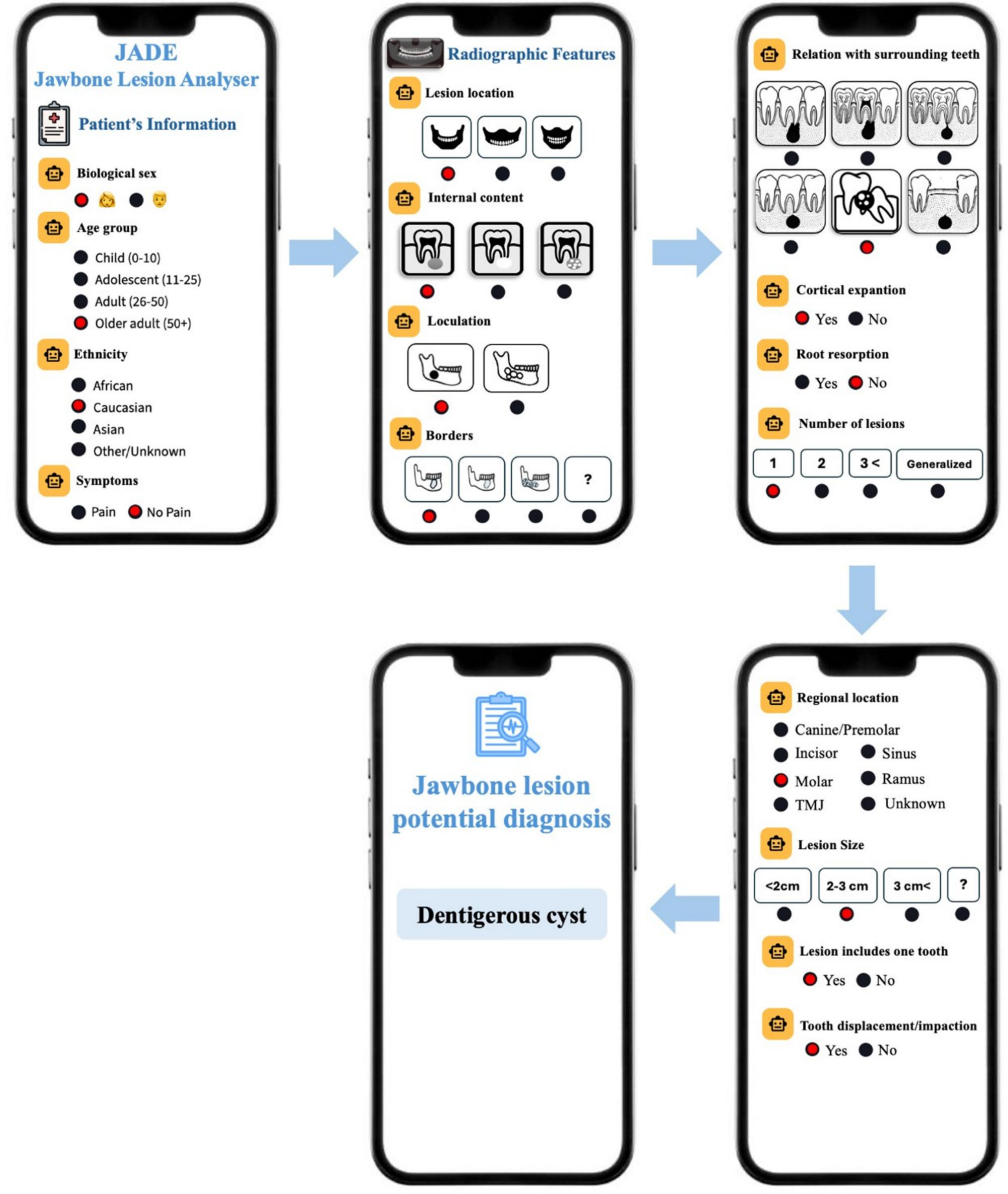
The figure presents how the patient’s information and radiographic characteristics are sequentially entered into JADE to generate a diagnosis for a representative case. In this example, the patient is a 53-year-old Caucasian female with no pain symptoms. The lesion is located in the mandibular molar region, linked to the crown, and presented as a single lesion. Radiographic features include a 2-3 cm size, central origin, and unilocular appearance, with corticated borders. The lesion involves multiple teeth, causes cortical expansion and tooth displacement, but no sign of root resorption. Based on these structured inputs, JADE correctly predicts a dentigerous cyst, matching the histopathological diagnosis.

The end-to-end diagnostic workflow of the JADE application is presented in Figure 3, showing how the system transforms structured patient data and lesion features into a final predicted diagnosis for a representative validation case.

**Figure 4:**
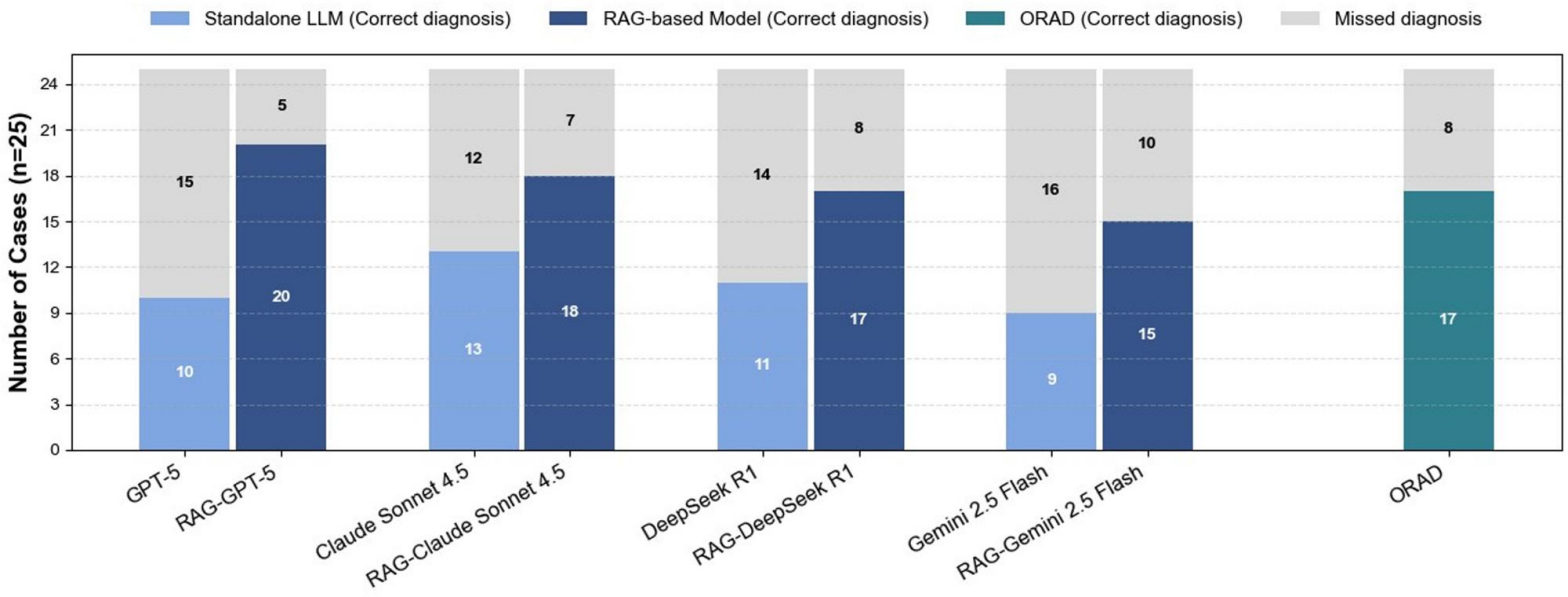
Comparative diagnostic performance across 25 validation cases (*n* = 25) for standalone LLMs, their corresponding RAG implementations, and the ORAD system. For each backbone model (GPT-5, Claude Sonnet 4.5, DeepSeek R1, and Gemini 2.5 Flash), diagnostic accuracy is presented as the number of correct and missed diagnoses. Integration of the RAG module consistently improves performance compared to standalone LLMs, demonstrating the benefit of knowledge-augmented reasoning. Among the evaluated systems, RAG-enhanced models achieve higher diagnostic accuracy overall, while ORAD shows competitive performance relative to several standalone configurations.

Across the 25 validation cases, RAG-enhanced configurations consistently achieved higher diagnostic accuracy than their corresponding standalone LLM backbones (Figure 4). Between all evaluated models, RAG-GPT-5 resulted in the highest number of correct diagnoses (20/25), followed by RAG-Claude Sonnet 4.5 (18/25), RAG-DeepSeek R1 (17/25), and RAG-Gemini 2.5 Flash (15/25). In contrast, standalone models showed lower performance, with Claude Sonnet 4.5 achieving the highest number of correct diagnoses (13/25), GPT-5 showing intermediate performance with 10 correct diagnoses, and Gemini 2.5 Flash demonstrating the lowest performance (9/25). The supervised ORAD system achieved 17 correct diagnoses out of 25 cases.

**Figure 5:**
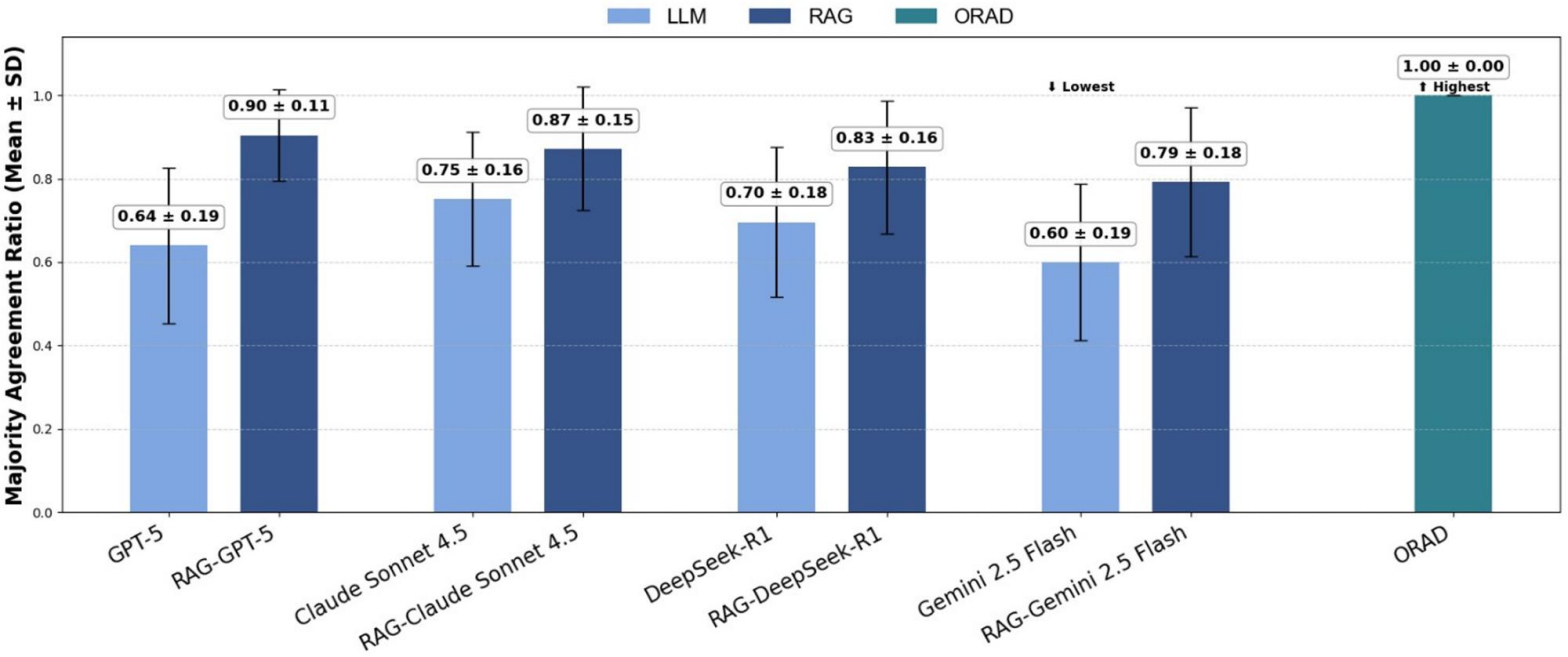
Comparison of intra-model stability across standalone LLMs, their corresponding RAG-enhanced configurations, and the ORAD system, expressed as MAR (mean ± SD) over repeated inference runs. For each LLM backbone (GPT-5, Claude Sonnet 4.5, DeepSeek R1, and Gemini 2.5 Flash), RAG integration is associated with higher stability compared to the respective standalone model. Among the LLM-based systems, RAG-GPT-5 demonstrated the highest stability, while greater variability is observed among standalone configurations.

The intra-model stability measured using the majority agreement ratio (MAR; mean ± SD) showed variability across configurations (Figure 5). Among standalone models, Claude Sonnet 4.5 achieved the highest MAR (0.75 ± 0.16), followed by DeepSeek R1 and GPT-5, while Gemini 2.5 Flash showed the lowest MAR (0.60 ± 0.19). The corresponding RAG-enhanced configurations demonstrated higher MAR values across all backbones, with RAG-GPT-5 achieving the highest MAR (0.90 ± 0.11), followed by RAG-Claude Sonnet 4.5 (0.87 ± 0.15), while RAG-Gemini 2.5 Flash showed the lowest MAR (0.79 ± 0.18). The ORAD system showed the highest stability among all evaluated systems.

Cochran’s *Q* test showed no statistically significant difference in diagnostic performance between the 4 standalone LLMs (*Q*(3) = 3.89, *P *= .27). Subsequent pairwise comparisons using McNemar’s exact test, with Bonferroni correction applied for multiple testing (adjusted *α* = .008), revealed no statistically significant differences between any standalone model pairs.

Similarly, Cochran *Q* test demonstrated no statistically significant difference among the RAG-enhanced models and ORAD (*Q*(4) = 4.71, *P *= .31). *Post hoc* pairwise McNemar exact tests, adjusted using Bonferroni correction (adjusted *α* = .005), showed no statistically significant differences between any RAG or ORAD model combinations.

Direct paired comparisons between each standalone LLM and its corresponding RAG-enhanced configuration were performed using McNemar exact test, with Bonferroni correction applied (adjusted *α* = .0125). A statistically significant improvement was observed for GPT-5 when integrated with the RAG framework (*P *= .002). No statistically significant differences were detected between standalone and RAG configurations for Gemini 2.5 Flash (*P *= .109), DeepSeek R1 (*P *= .031), or Claude Sonnet 4.5 (*P *= .063), as all *P*-values exceeded the corrected significance threshold.

Furthermore, response time analysis showed variability between the RAG-based configurations. RAG-DeepSeek R1 demonstrated the longest latency, ranging from 7 ± 0.4 to 10 ± 0.7 s, whereas RAG-Gemini 2.5 Flash exhibited the shortest latency, ranging from 3 ± 0.3 to 6 ± 0.5 s. RAG-GPT-5 had response times between 6 ± 0.4 and 8 ± 0.6 s, while RAG-Claude Sonnet 4.5 showed response times ranging from 6 ± 0.4 to 9 ± 0.6 s.

## Discussion

This study introduced JADE, a novel retrieval-augmented diagnostic framework designed to enhance differential diagnosis of jawbone lesions by combining structured clinical inputs, domain-specific knowledge retrieval, and LLM-based reasoning within an end-to-end diagnostic system. Instead of relying only on the general knowledge embedded within a standalone LLM or exclusively on static domain references, JADE included both sources through a RAG pipeline. Using a hybrid search strategy that combined keyword and semantic-based search, the system retrieved validated radiological information from an expert-curated jawbone lesion database and integrated this evidence into the structured prompt before response generation with the LLM. This pipeline allowed JADE to generate clinically relevant diagnostic predictions, overcoming the limitations of standalone LLMs that rely on patterns learned during training. Moreover, JADE was developed as a cloud-based, mobile-adapted system, enhancing accessibility for general dentists, oral radiologists, and dental trainees.

The integration of hybrid retrieval substantially improved both diagnostic performance and intra-model stability. Standalone LLMs rely completely on internal representations learned during pretraining, which may result in variability over repeated inference runs and inconsistent prioritization of radiographic features. In contrast, the RAG configuration, in which reasoning was based on retrieved case-relevant domain knowledge, limited generative variability. The significantly higher intra-model stability observed in the RAG configurations indicated that retrieval grounding reduced random variation and improved reproducibility. Such stability made the proposed system clinically relevant since reproducible outputs reduced ambiguity and strengthened confidence in the AI-assisted decision-making process.

Statistical analyses further supported that retrieval augmentation improved diagnostic accuracy compared to standalone LLMs. Although standalone Claude Sonnet 4.5 and DeepSeek-R1 achieved superior or competitive raw performance compared to standalone GPT-5, the RAG-enhanced GPT-5 model showed considerably improved accuracy and intra-model stability.

Importantly, the retrieval pipeline, including the embedding model, hybrid dense-sparse search strategy, and indexed domain-specific database, was identical across all evaluated RAG configurations. Therefore, observed performance differences cannot be attributed to variations in retrieval quality but rather to differences in how effectively each LLM used the retrieved contextual evidence during response generation. This finding indicated that diagnostic reliability within a RAG framework depended not only on the quality of retrieval but also on the model’s capacity to interpret, integrate, and reason over externally supplied domain-specific information.

In comparison with ORAD, a supervised learning-based diagnostic system, the highest stability across repeated runs was observed, as expected for deterministic supervised models. However, its diagnostic accuracy did not surpass that of the RAG-enhanced configuration. In this study, only the primary prediction (ie, the first-ranked diagnosis) was considered for statistical comparison. Under this evaluation criterion, the RAG-GPT-5 system particularly demonstrated superior performance. These findings suggested that although supervised models currently offer greater stability and established performance, RAG systems demonstrated acceptable capability. With further refinement and optimization, RAG-based systems may progressively approach the performance level of supervised methods in specialized diagnostic settings.

While GPT-5 did not consistently outperform other models in standalone mode, its integration within the RAG framework resulted in stable and clinically coherent outputs. GPT-5 consistently followed the provided instructions and reliably incorporated retrieved evidence into the final differential diagnosis. In contrast, models that performed strongly in standalone settings may rely more heavily on internal representations and may not integrate external contextual evidence as systematically when operating within a retrieval-augmented pipeline. Thus, the superior performance of the RAG-enhanced GPT-5 configuration suggested that generation quality within context-augmented settings was a critical determinant of diagnostic improvement.

Based on these findings, GPT-5 was selected for continued system development due to its balanced performance within the RAG environment, where proper use of contextual information, reasoning consistency, and output stability are necessary for clinical applicability. Moreover, OpenAI’s GPT series has demonstrated rapid and continuous development since the public release of ChatGPT in 2022,^37^ showing rapid development cycles and model improvements within the broader LLM ecosystem. This pattern supports its suitability as a stable generative backbone for future system expansion.

In addition, JADE achieved a mean end-to-end response time of 6 s. This included both the hybrid retrieval step (which added only a minor overhead of <0.5 s) and GPT-5 reasoning. The overall latency was comparable to, and in some cases faster than, standalone LLMs’ responses (typically 4-6 s), indicating that the addition of the RAG component did not substantially increase response time. Among the evaluated configurations, the RAG-Gemini model showed the shortest response time, likely reflecting faster generative processing; however, this reduction in latency resulted in lower diagnostic performance. In contrast, the RAG-enhanced GPT-5 configuration achieved a balanced trade-off between response time, diagnostic accuracy, and stability. As a mobile-accessible cloud platform, JADE therefore operated within the range required for rapid clinical decision support and educational use.

These findings are aligned with recent studies evaluating LLM models in oral diagnosis. Kaygisiz et al.^9^ reported that DeepSeek-v3 showed moderate diagnostic accuracy and performed better than GPT-4o, with scores of 4.02 ± 0.36 vs 3.15 ± 0.4, and outperformed GPT-4o in 9 of 16 simulated cases However, both models showed limited real-world applicability, as they failed to correctly diagnose almost half of the representative cases, highlighting the limitations faced by standalone LLMs in complex diagnostic scenarios. Hassanein et al.^11^ found Top-1 accuracy on 80 clinical cases, with DeepSeek-V3 achieving 45% and GPT-4o 40% and only reaching 70% accuracy at the Top-3. Zhuang et al.^38^ further reported diagnostic accuracy of 71% for Claude Sonnet 3.5 and 57% for GPT-4o, consistent with our finding in which Claude Sonnet outperformed GPT-based models. In another study, Pradhan et al.^39^ found that GPT-4o correctly diagnosed 28 out of 42 potentially malignant lesions. Whereas Gemini demonstrated substantially weaker performance with only 15 correct diagnoses. Similarly, Kim et al.^10^ showed that ChatGPT-4 achieved a concordance rate of 41.4%, comparable to clinicians (43.2%) and slightly below the ORAD system (45.6%). Overall, these results confirmed the inconsistent and often limited diagnostic reliability of standalone LLMs, a pattern also reflected in our findings and underscoring the added value of retrieval-augmented systems such as JADE.

Despite its strengths, this study has several limitations. The system depends on the accuracy of structured inputs provided by clinicians and currently lacks automated input validation, which may introduce variability. Future developments should aim to reduce this manual dependency. In addition, the current system incorporates only limited patient information, which may restrict the depth of clinical context and consequently influence diagnostic precision. Furthermore, a limited number of cases were misdiagnosed, suggesting that the RAG mechanism and hybrid retrieval strategy alone cannot fully eliminate diagnostic errors. Future advancements may integrate an agentic self-evaluating mechanism able to automatically re-evaluate or refine the predicted outcome against the key clinical and radiographic parameters provided in the query. The domain-specific database could also be optimized by reconstructing it into shorter, parameter-specific documents to allow more precise retrieval that can be dynamically controlled by an agentic mechanism. Another limitation of this study is the relatively small validation dataset that limits the generalizability of the system. Future studies should overcome these challenges and prioritize expanding the dataset to include a wider range of jawbone lesions.

## Conclusion

This study introduced JADE as a proof-of-concept RAG system for the differential diagnosis of jawbone lesions. By integrating structured clinical input with hybrid knowledge retrieval and generative reasoning, the system demonstrated improved diagnostic accuracy and intra-model stability compared to standalone LLMs, while maintaining a rapid response time clinically. Although supervised models such as ORAD demonstrated higher deterministic stability, the RAG-based configuration achieved competitive primary diagnostic performance and provided adaptive contextual reasoning beyond fixed classification boundaries. Importantly, this study further demonstrated the feasibility and clinical relevance of incorporating a domain-specific RAG framework into dentomaxillofacial, an approach that has not previously been assessed in this field. While further improvements are required to improve the generalizability, continued development, and wider validation, this pipeline may contribute to more accurate, consistent, and accessible diagnostic assistance within oral and maxillofacial radiology.

## Supplementary Material

twag017_Supplementary_Data
