# Supplementary material for: JADE: jawbone lesion diagnosis and decision supporting system
Source: Dentomaxillofac Radiol. 2026 Mar 23;55(5):497–507. doi: 10.1093/dmfr/twag017 (PMC13317976; doi:10.1093/dmfr/twag017)
Supplement: twag017_Supplementary_Data [file twag017_supplementary_data.pdf]

Non-odontogenic bone lesions

1. Osseous lesions

1.1. Benign osseous lesions

1.1.1. Exostoses and torus palatinus/mandibularis

Exostoses and Tori Overview

- Benign bony outgrowths in the oral cavity.
- Characterized by slow growth and are generally asymptomatic.

Prevalence and Epidemiology

- **Torus Palatinus**
  - Prevalence: ~20%, making it the most common form of hyperostosis.
  - Gender preference: Occurs approximately twice as often in women compared to men.
  - More common among Indigenous Americans, Eskimos, and Norwegians.
  - Rare in children; typically develops in young adults before age 30.
- **Torus Mandibularis**
  - Prevalence: ~8%.
  - Can be single or multiple, unilateral or bilateral (most often bilateral).
  - Most commonly located in the premolar region.
  - Gender preference: More frequent in women.
  - Correlation: In women, often occurs alongside torus palatinus, but this association is not observed in men.
  - Develops later than torus palatinus, typically detected in middle-aged adults.
- **Exostoses**
  - Less common than tori.

Clinical Symptoms

- **Typically asymptomatic.**
- **Patients may present with a hard, painless swelling in the affected area.**

Radiographic Appearance on Panoramic Imaging

- **Exostoses**
  - **Peripheral:**
    - Well-defined radiopacity with bone density arising from cortical plates.
    - May be pedunculated or sessile with a broad base.
  - **Central:**
    - Originate from endosteal surfaces of the bone.
    - No radiolucent halo; May cause bone expansion or displacement of adjacent teeth.
- **Torus Palatinus**
  - Radiopaque area located in the midline of the hard palate.
  - Size and shape may vary.
- **Torus Mandibularis**
  - Bilateral, rounded, or nodular radiopacities along the lingual aspects of the mandible.
  - Typically located in the premolar region.

Radiological Differential Diagnosis

- **Idiopathic Osteosclerosis**
  - Small, round, well-defined radiopacity without a radiolucent halo.
- **Sclerosing Osteitis**
  - Associated with a non-vital tooth, unlike exostoses and tori.
- **Osteoma**

    - Larger than exostoses or tori. May present with a more irregular shape.
- **Complex Odontoma**
    - Exhibits a more irregular radiopaque pattern compared to exostoses and tori.
- **Cementoblastoma**
    - Typically attached to the root of a tooth with a radiolucent halo.
- **Osteoblastoma**
    - Larger than osteomas, occasionally cause pain.
- **Hypercementosis**
    - Appears as a thickening of the tooth root.

1.1.2. Idiopathic osteosclerosis / dense bone island
1.1.3. Osteoma
1.1.4. Multiple osteomas in Gardner syndrome
1.1.5. Osteoid osteoma
1.1.6. Osteoblastoma

1.2. Malignant osseous lesions

1.2.1. Osteosarcoma
1.2.2. Metastasis to the jawbones

2. Chondroid lesions

2.1. Benign chondroid lesions

2.1.1. Chondroma
2.1.2. Osteochondroma
2.1.3. Chondroblastoma

2.2. Malignant chondroid lesions

2.2.1. Chondrosarcoma

3. Fibro-osseous lesions

3.1. Fibrous dysplasia

3.2. Ossifying fibroma

3.2.1. Cemento-ossifying fibroma
3.2.2. Juvenile trabecular ossifying fibroma
3.2.3. Psammomatoid ossifying fibroma

3.3. Cemento-osseous dysplasia

3.3.1. Periapical cemento-osseous dysplasia
3.3.2. Focal cemento-osseous dysplasia
3.3.3. Florid cemento-osseous dysplasia
3.3.4. Familial gigantiform cementoma / expansive cemento-osseous dysplasia

3.4. Segmental odontomaxillary dysplasia

4. Fibrous bone lesions

4.1. Desmoplastic fibroma of bone

4.2. Desmoid fibromatosis

4.3. Myofibromatosis

4.4. (Solitary) myofibroblastoma / myofibroma

5. Giant cell lesions

5.1. Central giant cell granuloma

5.2. Aneurysmal bone cyst

5.3. Cherubism
5.4. Giant cell lesions in hyperparathyroidism (Brown tumor)

6. Vascular bone lesions
6.1. Central hemangioma

7. Hematopoietic bone lesions
7.1. Langerhans cell histiocytosis
7.2. Multiple myeloma
7.3. Solitary plasmacytoma
7.4. Lymphoma

8. Neurogenic and neuro-ectodermal lesions
8.1. Central schwannoma
8.2. Central neurofibroma
8.3. Ewing sarcoma

9. Soft tissue opacities
9.1. Tonsilloliths
9.2. Calcified lymphadenopathy
9.3. Anthroliths
9.4. Sialoliths
9.5. Calcified stylohyoid ligament
9.6. Calcified carotid artery

Odontogenic tumors

1. Benign odontogenic tumors
1.1. Epithelial odontogenic tumors
- 1.1.1. Conventional ameloblastoma
- 1.1.2. Unicystic ameloblastoma
- 1.1.3. Calcifying epithelial odontogenic tumor (Pindborg tumor)
- 1.1.4. Squamous odontogenic tumor
- 1.1.5. Adenomatoid odontogenic tumor

1.2. Mixed epithelial–ectomesenchymal odontogenic tumors
- 1.2.1. Ameloblastic fibroma
- 1.2.2. Ameloblastic fibro-odontoma
- 1.2.3. Odontoma
- 1.2.4. Dentinogenic ghost cell tumor
- 1.2.5. Primordial odontogenic tumor

1.3. Ectomesenchymal odontogenic tumors
- 1.3.1. Central odontogenic fibroma
- 1.3.2. Odontogenic myxoma
- 1.3.3. Cementoblastoma
- 1.3.4. Cemento-ossifying fibroma

2. Malignant odontogenic tumors

2.1. Odontogenic carcinoma
2.2. Odontogenic sarcoma

Cysts of the jawbones

1. Developmental cysts
1.1. Odontogenic developmental cysts
1.1.1. Dentigerous cyst
1.1.2. Odontogenic keratocyst
1.1.3. Orthokeratinized odontogenic cyst
1.1.4. Lateral periodontal cyst
1.1.5. Botryoid odontogenic cyst
1.1.6. Glandular odontogenic cyst
1.1.7. Calcifying odontogenic cyst (Gorlin cyst)
1.2. Non-odontogenic developmental cysts
1.2.1. Nasopalatine duct cyst
1.2.2. Surgical ciliated cyst

2. Inflammatory cysts
2.1. Periapical inflammatory cystic lesions
2.1.1. Periapical granuloma
2.1.2. Radicular cyst
2.1.3. Residual cyst
2.1.4. Post-treatment periapical scar
2.2. Inflammatory collateral cysts
2.2.1. Mandibular buccal bifurcation cyst
2.2.2. Distal paradental cyst

3. Pseudocysts
3.1. Anatomical variations
3.1.1. Stafne bone defect
3.1.2. Focal osteoporotic bone marrow defect
3.2. Reactive and idiopathic pseudocysts
3.2.1. Simple bone cavity
3.2.2. Pseudotumor in hemophilia

Infectious and inflammatory bone pathology

1. Osteomyelitis
1.1. Acute osteomyelitis
1.2. Chronic osteomyelitis
1.2.1. Focal sclerosing osteomyelitis (condensing osteitis)
1.2.2. Diffuse sclerosing osteomyelitis
1.2.3. Chronic osteomyelitis with proliferative periostitis (Garré’s osteomyelitis)
2. Osteonecrosis

2.1. Medication-related osteonecrosis of the jaw
2.2. Osteoradionecrosis

3. Periodontitis
3.1. Localized periodontitis
3.2. Diffuse periodontitis

Systemic diseases

1. Paget's disease
2. Infantile cortical hyperostosis (Caffey's disease)3. Vanishing bone disease (Gorham–Stout disease)
